# Copper Chaperone for Superoxide Dismutase Promotes Breast Cancer Cell Proliferation and Migration *via* ROS-Mediated MAPK/ERK Signaling

**DOI:** 10.3389/fphar.2019.00356

**Published:** 2019-04-05

**Authors:** Yanping Li, Ronghui Liang, Xiaoya Zhang, Jiyan Wang, Changliang Shan, Shuangping Liu, Leilei Li, Shuai Zhang

**Affiliations:** ^1^ Biomedical Translational Research Institute, Jinan University, Guangzhou, China; ^2^ State Key Laboratory of Medicinal Chemical Biology, College of Pharmacy and Tianjin Key Laboratory of Molecular Drug Research, Nankai University, Tianjin, China; ^3^ Department of Pathology, Medical School, Dalian University, Dalian, China; ^4^ School of Integrative Medicine, Tianjin University of Traditional Chinese Medicine, Tianjin, China

**Keywords:** breast cancer, CCS, ROS, MAPK/ERK, proliferation, migration

## Abstract

Copper chaperone for superoxide dismutase (CCS) is a critical component of oxidation–reduction system and functions as a potential tumor promoter in several cancers. However, the function and clinical significance of CCS in breast cancer remain unclear. Here, we found CCS was highly expressed in breast cancer, where it promoted breast cancer cell proliferation and migration. Suppression of CCS expression was sufficient to attenuate the phosphorylation level of ERK1/2 and increase the accumulation of reactive oxygen species (ROS). Mechanistically, we found that knockdown of CCS decreases the activity of ERK1/2 mediated by the accumulation of ROS, which leads to the inhibition of cell proliferation and migration. In summary, these results indicated that CCS promotes the growth and migration of breast cancer cells *via* regulating the ERK1/2 activity mediated by ROS.

## Introduction

Breast cancer is the leading cause of cancer-related deaths in women worldwide (Christofori, 2006; Bray et al., 2018). Breast cancer patients with metastases have an extremely poor prognosis (Gupta et al., 2005; Bacac and Stamenkovic, 2008; Thiery, 2009; Chaffer and Weinberg, 2011; Valastyan and Weinberg, 2011). Thus, exploring new targets for breast cancer treatment is important.

Copper, a redox-active transition metal essential for most living organisms, serves as a catalytic cofactor for enzymes that function in antioxidant defense, iron homeostasis, cellular respiration, and a variety of biochemical processes (Mandinov et al., 2003; Lowndes and Harris, 2005; Ashino et al., 2010; Xu et al., 2016; Sciegienka et al., 2017). The uncontrolled accumulation of copper could lead to increased oxidative stress and inappropriate binding to macromolecules. Copper chaperone for superoxide dismutase (CCS) delivers copper to specific cellular destinations and to superoxide dismutase (SOD1) (Kawamata and Manfredi, 2008; Ulloa, 2009). Mounting evidences suggest that CCS plays a crucial role in oxidative metabolism (Kawamata and Manfredi, 2008; Leitch et al., 2009; Suzuki et al., 2013a; Wang et al., 2015). Blockade of the copper-trafficking chaperone CCS contributes to the increased cellular reactive oxygen species (ROS) level due to the overall accumulation of copper inside the cells and the decreased SOD1 activity (Ulloa, 2009). Wang et al reported that inhibiting CCS blocks lung cancer and leukemia cell growth (Wang et al., 2015). In addition, they show that blocking copper trafficking induces cellular oxidative stress and reduces cellular ATP levels. The reduced level of ATP results in activation of the AMP-activated protein kinase that leads to reduced lipogenesis. However, the mechanisms underlying the relationship between CCS and tumorigenesis are still largely unknown, although the positive correlation between CCS and redox homeostasis has been revealed (Wang et al., 2015). Therefore, this study aimed to explore the critical role and molecular mechanism of CCS in migration and proliferation of breast cancer.

In aforementioned study by Wang et al, a CCS inhibitor was developed and shown to have the same effect as knocking down CCS in cancer cells (Wang et al., 2015). However, the precise role of CCS in migration and proliferation of breast cancer cells is unknown. In the present study, we report that CCS is highly expressed in breast cancer tissues and invasive breast cancer cells and promotes cell proliferation and migration. Furthermore, we found that inhibition of CCS by shRNA or an inhibitor blocks breast cancer proliferation and migration by triggering ROS mediated ERK activity. These results suggest that metastasis-prone breast cancer cells reprogram oxidative metabolism to promote cell proliferation and migration. Targeting CCS may represent a promising approach for selectively causing cell proliferation and migration in breast cancer cells.

## Materials and Methods

### Reagents and Antibodies

DC_AC50, a CCS inhibitor, was provided by the Shanghai Institute of Materia Medica of the Chinese Academy of Sciences. U0126-EtOH (catalog number: S1102) was purchased from Selleck. Antibody against phospho-p44/42 MAPK (Erk1/2) (Thr202/Tyr204) (1:1000 times dilution) (catalog number: 4370S), p44/42 MAPK (Erk1/2) (1:1000 times dilution) (catalog number: 4695S), phpspho-MEK1/2 (Ser217/221) (1:1000 times dilution) (catalog number: 9154S), MEK1/2 (1:1000 times dilution) (catalog number: 8727S), β-actin (1:1000 times dilution) (catalog number: 8457S), mouse IgG (1:3000 times dilution) (catalog number: 7076S), and rabbit IgG (1:3000 times dilution) (catalog number: 7074S) were from cell signaling technology. Anti-Superoxide Dismutase 4 (1:500 times dilution) (catalog number: ab167170) was from Abcam. Anti-Flag tag (1:1000 times dilution) (catalog number: 66008) was from proteintech. CCS shRNA was purchased from Open Biosystems, Huntsville, AL. The sequence of targeted CCS shRNA was as follows: 5′-CCGGCTGATTATTGATGAGGGAGAACTCGAGTTCTCCCTCATCAATA ATCAGTTTTTG-3′. Lipofectamine RNA iMAX was purchased from Invitrogen. The sequences of targeted CCS siRNA were as follows: sense: 5′-GUCUUGGUACACACCACUCUA-3′; Antisense: 5′-UAGAGUGGUGUGUACCAAGAC-3′.

### Cell Culture and Cell Lines

The human breast cancer cell lines MDA-MB-231, MCF-7, SUM159, and T47D were obtained from American Type Culture Collection (Manassas, USA). The human normal epithelial lung cell line BEAS-2B was gifted from Dr. Chenglai Xia (Guangzhou Medical University, Guangdong, China). MDA-MB-231, MCF-7, SUM159, and T47D cells were cultured in Dulbecco Modified Eagle Medium (DMEM) with 10% fetal bovine serum (FBS, ExCell Bio). BEAS-2B cells were cultured in RPMI 1640 medium with 10% FBS. For routine passages, cultures were split 1:3 when they reached 80–90% confluences. All experiments were performed on exponentially growing cells.

### Plasmid Construction and Lentivirus Packaging

Exogenous human CCS CDS sequence was inserted into pLVX-3FLAG plasmid. Primer sequences were as follows: pLVX-3FLAG-CCS: 5′-CGGGATCCATGGCTTCGGATTCGG-3′ (forward) and 5′-CCCTCGAGTCAAAGGTGGGCAGG-3′ (reverse). Exogenous pCDH-HA-MEK plasmid was gifted from Dr. ShiZhi (JinanUniversity, Guangdong, China). For transient transfections, cells were grown to 80% confluency and transfected with plasmids using PEI Transfection Reagent (Invitrogen, USA) according to the manufacturer’s protocol. Stable knockdown of endogenous CCS was achieved by using lentiviral vector harboring shRNA construct. 5′-CCGGCTGATTATTGATGAGGGAGAACTCGAGTTCTCCCTCATCAATAATCAGTTTTTG-3′. We generated CCS stable knockdown cell lines by infected lentiviral shRNA and selected by antibiotic puromycin. The knockdown effective was confirmed by western blot. PLKO.1 is the name of the lentiviral vector as a control.

### Small Interference RNA Transfection

MDA-MB-231, MCF-7, and BEAS-2B cells (2 × 10^5^) were seeded into 6-well plates and cultured in a humidified incubator at 37°C and 5% CO_2_ for 24 h. Cells were transfected with a negative control siRNA (NC-siRNA) and siRNA targeting CCS by Lipofectamine RNA iMAX (Invitrogen corporation). Transfected cells were cultured for 48°C before being used for further experiments. The sequences of targeted CCS siRNA were 5′-GUCUUGGUACACACCACUCUA-3′. The sequences of negative control siRNA were 5′-UUCUCCGAACGUGUCACGUTT-3′(forward). All siRNA sequences were purchased from the Invitrogen Ribobio corporation of Guangzhou.

### Real-Time Quantitative Reverse Transcription-PCR

Total cellular RNA was extracted using the Eastep & Super RNA Extract reagent Kit (Promega). cDNA was generated from purified RNA using PrimeSciptTM RT reagent Kit (Takara) according to the manufacturer’s instructions. Gene expression levels and PCR efficiency, along with its standard error, were calculated using the Bio-Rad CFX Manager, version 3.1 (Bio-Rad), The efficiencies were nearly 100%, allowing the use of the 2^−△△Ct^ method for calculating the relative gene expression levels and reference gene normalization using β-actin. All PCR runs were performed in triplicate, and the data analyzed by CFX Manager software (Bio-Rad). Primer sequences were as following: CCS: 5′-CATCGAGGGAACTATTGACG-3′ (forward) and 5′-ATGCTCCATCAGGGTTAAAG-3′(reverse); β-actin:5′-ACGTGGACATCCGCAAAG-3′ (forward) and 5′-GACTCGTCATACTCCTGCTTG-3′ (reverse).

### Cell Proliferation Assay

Cell proliferation assays were performed by seeding 5 × 10^4^ cells in 6-well plates and culturing the cells at 37°C. Relative cell proliferation was determined by cell numbers recorded at 4 days after being seeded and normalized to that of each of the cell lines at the starting time (t = 0 h).

### Western Blot Analysis

Cells were lysed with lysis buffer (1.5 M NaCl, 1 M HEPES [pH = 7.0], 1% NP40, 0.1 M Na_4_P_2_O_7_, 0.1 M NaF, 0.1 M Na_3_VO_4_, protease inhibitor) on ice 30 min and then centrifuged at 12,000 rpm for 15 min at 4°C. Protein samples were separated by 12%.

SDS-PAGE and transferred onto PVDF membranes (Millipore). The membranes were blocked with 5% non-fat milk for 2 h and then incubated overnight at 4°C with the primary antibody and 1 h at room temperature with secondary antibody. Signals were detected using luminol substrate solution.

### Transwell Migration Assay

For the Transwell (24-well insert, 8 mm pore size with polycarbonate membrane; Corning Costar, Lowell, MA, USA) migration assays, 600-mL media supplemented with 10% FBS was added to the lower chamber, and the cells resuspended in serum-free media were added to the upper insert after transfection. Transwell membranes were fixed and stained using crystal violet after specified time. The cells adhering to the lower surface of the membrane were counted under a light microscope (Olympus, Tokyo, Japan) at a magnification of 200.

### Wound Healing Assay

To determine cell motility, cells were seeded into 6-well plates and grown to 90% confluence. A monolayer of the cells was then scratched with a sterile micropipette tip, followed by washing with PBS to remove cellular debris. The cell migration was observed and counted under a light microscope (Olympus, Tokyo, Japan) at a magnification of 200. The cells that migrated across the black lines were counted in three randomly chosen fields from each triplicate treatment.

### Intracellular Reactive Oxygen Species (ROS) Production

The amount of intracellular ROS was measured by detecting dichlorodihydrofluorescein, which is the cleavage product of carboxy-H_2_DCFDA (Invitrogen) by ROS. A total of 200,000 cells were seeded in 6-well plate. Twenty-four hours after seeding, cells were washed with PBS and loaded with 12.5 μM carboxy-H_2_DCFDA for 60 min. The cells were harvested, resuspended in PBS, and analyzed using a FACS (BD Biosciences; excitation and emission at 490 and 530 nm, respectively).

### Bioinformatics Analysis

The public Gene Expression Omnibus datasets (GSE9574 and GSE21422) and the TCGA (The Cancer Genome Atlas) dataset were used for bioinformatics analysis.

### Statistical Analysis

The concentration of DC_AC50 required to reduce cell proliferation by 50% (IC50) was determined graphically using the Dose-response-Stimulation function in GraphPad Prism7 (San Diego, CA, United States). Statistical analyses of the significance of differences between groups were performed using Student’s t-test with GraphPad Prism7. All data were obtained from three independent experiments performed in triplicate and were presented as the mean ± standard error. *p* < 0.05 was considered to indicate a statistically significant difference.

## Results

### Higher CCS Gene Expression in Breast Cancer Patients

**Figure 1:**
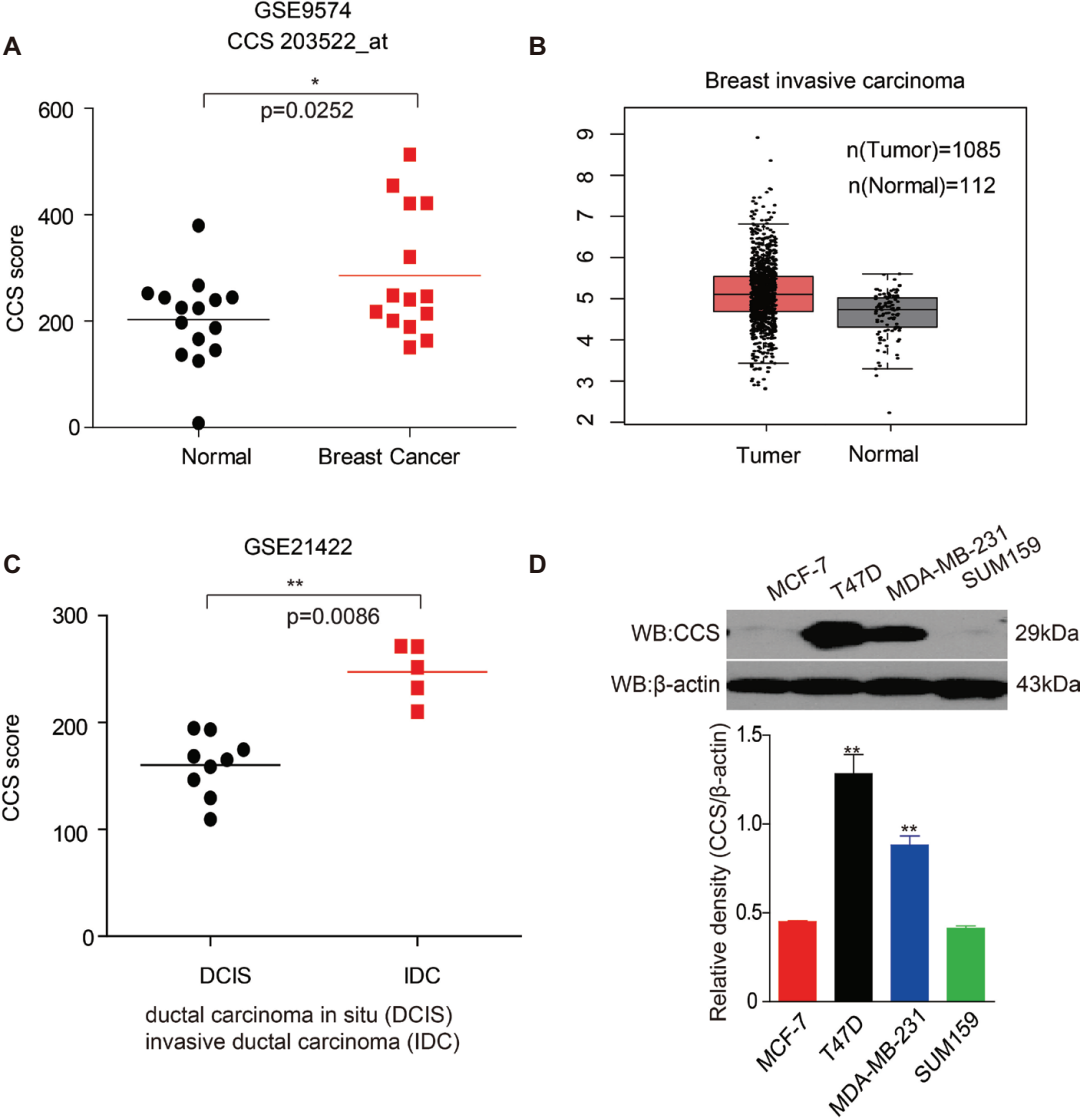
Up-regulation of CCS is associated with cell proliferation and metastasis in human breast cancer. **(A)** CCS expression was analyzed in normal and breast cancer cells using Gene Expression Omnibus (GEO) profiles (GSE9574). **(B)** CCS expression was determined in normal and breast cancer cells in TCGA. **(C)** CCS expression was analyzed in ductal carcinoma in situ (DCIS) and invasion ductal carcinoma (IDC) using Gene Expression Omnibus (GEO) profiles (GSE21422). **(D)** CCS protein levels were analyzed in the majority of a spectrum of diverse human breast cancer cells, including MCF-7, MDA-MB-231, SUM159, and T47D by western blotting. *p < 0.05; **p < 0.01.

Bioinformatics analysis has been used to discover previously unknown function of genes associated with cancer. To determine the role of CCS in human breast cancer, we first examined the expression of CCS utilizing Gene Expression Omnibus (GEO) profiles; we found that the expression of CCS was higher in breast cancer tissue than in noncancerous tissue (Figure 1A, GSE9574). We also confirmed these finding using The Cancer Genome Atlas (TCGA) dataset. CCS expression was also significantly higher in breast cancer tissue than in noncancerous tissue in the Cancer Genome Atlas (TCGA) (Figure 1B). In addition, we also found that the expression of CCS was higher in invasive ductal carcinoma (IDC) than in ductal carcinoma (DCIS) (Figure 1A, GSE21422). To validate these findings, we checked CCS expression in various breast cancer cells lines. CCS was differentially expressed in several breast cancer cell lines, including MCF-7, T47D, MDA-MB-231, and SUM159. Of note, the expression of CCS was higher in T47D and MDA-MB-231 cell lines compared to MCF-7 and SUM159 cells (Figure 1D). All these findings indicate the potential role of CCS in tumor formation and progression.

### CCS Promotes Breast Cancer Cell Proliferation *in vitro*

**Figure 2:**
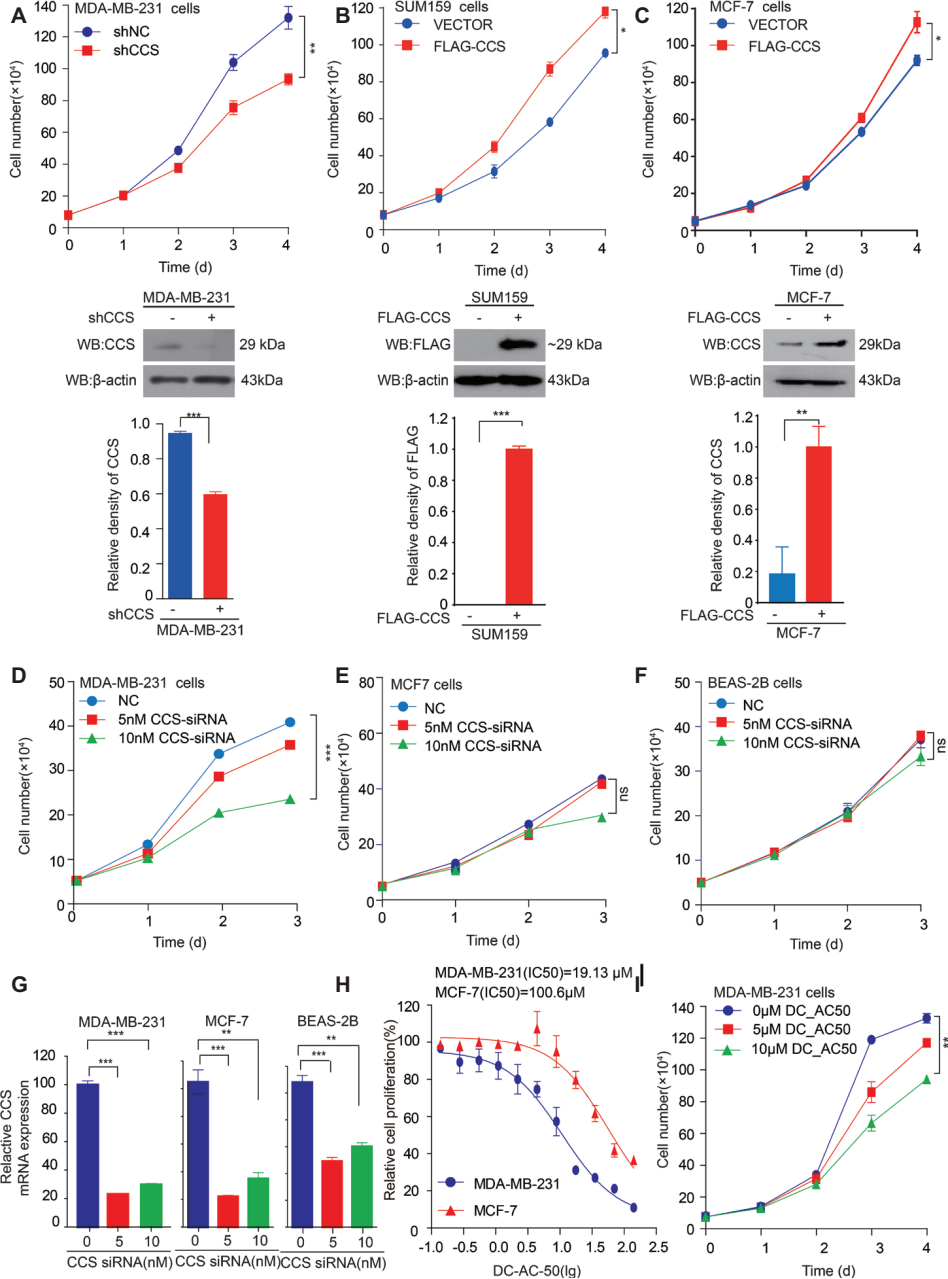
CCS promotes breast cancer cell proliferation. **(A)** Cell proliferation was determined by cell number counting assays in CCS stable knockdown MDA-MB-231 cells, and the knockdown efficiency was determined by western blotting. **(B)** Cell proliferation was determined by cell number counting assays in CCS overexpressing SUM159 cells, and CCS expression was determined by western blotting. **(C)** Cell proliferation was determined by cell number counting assays in CCS overexpressing MCF-7 cells, and CCS expression was determined by western blotting. **(D–F)** Cell proliferation was determined by cell number counting assays in MDA-MB-231 cells **(D)**, MCF-7 cells **(E)**, and normal human BEAS-2B cells **(F)**, which were transiently transfected with increasing concentrations of CCS siRNA and control siRNA. **(G)** The relative CCS mRNA level was determined by q-PCR in MDA-MB-231, MCF-7and BEAS-2B cells, which were transiently transfected with increasing concentrations of CCS siRNA and control siRNA. **(H)** The sensitivities of MDA-MB-231 and MCF-7 cells to DC_AC50 were determined by cell number counting assays when the cells were treated with increasing concentrations of DC_AC50 for 48 h. **(I)** Cell proliferation was determined by cell count assays in MDA-MB-231 cells treated with increasing concentrations of DC_AC50. All results performed above are presented as mean ± SD from three independent experiments. *p < 0.05; **p < 0.01; ***p < 0.001, ns: not significant.

We found that the expression of CCS was higher in breast cancer tissue than in noncancerous tissue, suggesting the potential role of CCS in breast cancer cell proliferation. To test our hypothesis, we generated stable cell lines in which CCS was knocked down in MDA-MB-231 cells (Figure 2A lower) and exogenously expressed in MCF-7 and SUM159 cells (Figures 2B,C lower). Cell number counting assays showed that knockdown of CCS reduced the proliferation of MDA-MB-231 cells (Figure 2A upper), while exogenous expression of CCS demonstrated the opposite effect (Figures 2B,C upper). To validate these findings, we knocked down the expression of CCS in MDA-MB-231, MCF-7, and BEAS-2B cells using siRNA. Cell number counting assays showed that knockdown of CCS significantly inhibited the proliferation of metastasis-prone breast cancer cell lines MDA-MB-231 but did not have any effect on the proliferation of breast cancer MCF-7 cells or normal BEAS-2B cells (Figures 2D–F). Real-time PCR was used to determine the knockdown efficiency of CCS by siRNA (Figure 2G). Next, we sought to explore the role DC_AC50, a potent and selective CCS inhibitor, in breast cancer. DC_AC50 has been shown to inhibit the proliferation of acute leukemia cells (Wang et al., 2015). We treated cells with DC_AC50 and found that MCF-7 cells exhibited significantly higher resistance to DC_AC50 than MDA-MB-231 cells (Figure 2H). Meanwhile, DC_AC50 treatment resulted in decreased cell proliferation of MDA-MB-231 cells in a time and dose-dependent manner (Figure 2I). These results imply that CCS plays an important role in breast cancer cell proliferation and suggests that CCS is a promising anti-cancer target.

### CCS Promotes Breast Cancer Cells Migration

**Figure 3:**
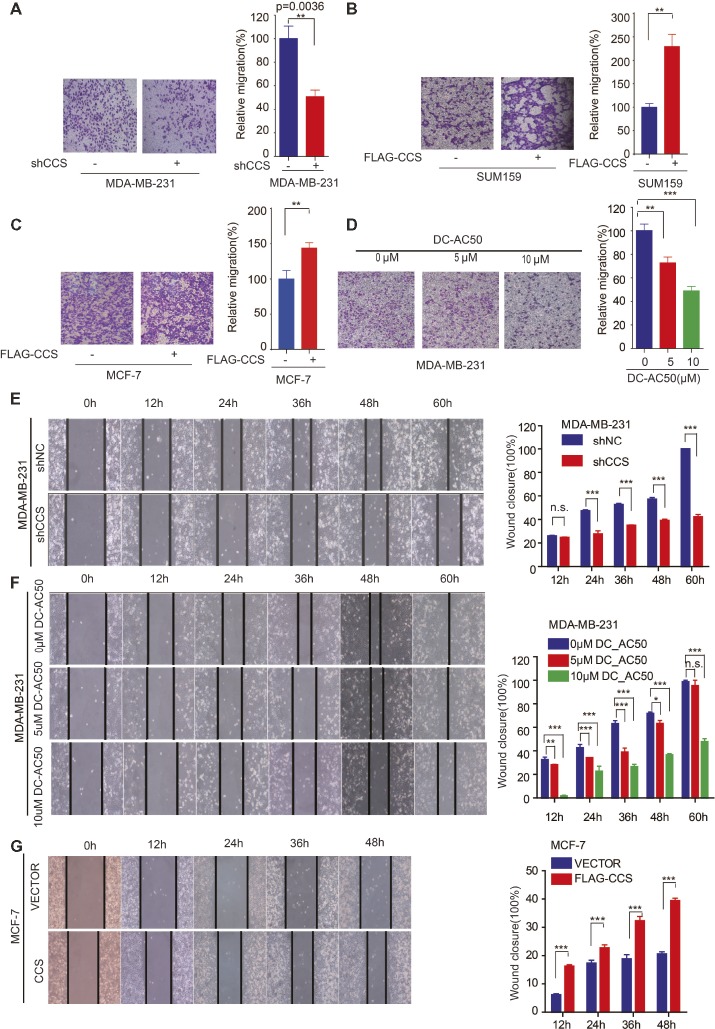
CCS promotes breast cancer cell migration. **(A)** Cell migration in CCS knockdown and control MDA-MB-231 cells was determined by transwell migration assay (Boyden chamber assay). **(B)** Cell migration in CCS overexpressing and control SUM159 cells was determined by transwell migration assay. **(C)** Cell migration in CCS overexpressing and control MCF-7 cells was determined by transwell migration assay. **(D)** Cell migration in CCS overexpressing and control MDA-MB-231 cells with increasing concentrations of DC_AC50 was determined by transwell migration assay. **(E)** Cell migration in CCS knockdown and control MDA-MB-231 cells was also determined by wound healing assay. **(F)** Cell migration in MDA-MB-231 cells treated with increasing concentrations of DC_AC50 was determined by wound healing assay. **(G)** Cell migration in CCS overexpressing and control MCF-7 cells was determined by the wound healing assay. The modified migration assay was evaluated by calculating the ratio of the cell numbers through the chamber or wound closure after the wound healing assay. All results performed above are presented as mean ± SD from three independent experiments. *p < 0.05; **p < 0.01; ***p < 0.001, ns: not significant.

We found that the expression of CCS was higher in invasive ductal carcinoma than in ductal carcinoma (Figure 1C), suggesting the potential role of CCS in promoting breast cancer migration. Next, we explore the role of CCS in the motility of the breast cancer cells. We performed a transwell migration assay that showed knockdown of CCS significantly inhibited breast cell migratory abilities in MDA-MB-231 (Figure 3A), while exogenous express CCS exhibited the opposite effects in MCF-7 and SUM159 cells (Figures 3B,C). To validate these finding, we treated MDA-MB-231 with CCS inhibitor, DC_AC50, and performed a transwell migration assay. We found that DC_AC50 blocked MDA-MB-231 cell migration in a dose-dependent manner (Figure 3D). In addition, we also assessed migration of MDA-MB-231 in a wound healing assay. We found that knockdown or inhibition of CCS dramatically suppressed MDA-MB-231 cell migratory abilities (Figures 3E,F). To consolidate our findings, we overexpressed FLAG tagged CCS in MCF-7 cells. As expected, overexpression of CCS accelerated breast cancer cell migration in a wound healing assay (Figure 3G). Taken together, our results suggest that CCS plays an important role in promoting breast cancer cells migration.

### CCS Promotes Breast Cancer Migration *via* MAPK/ERK Signaling

**Figure 4:**
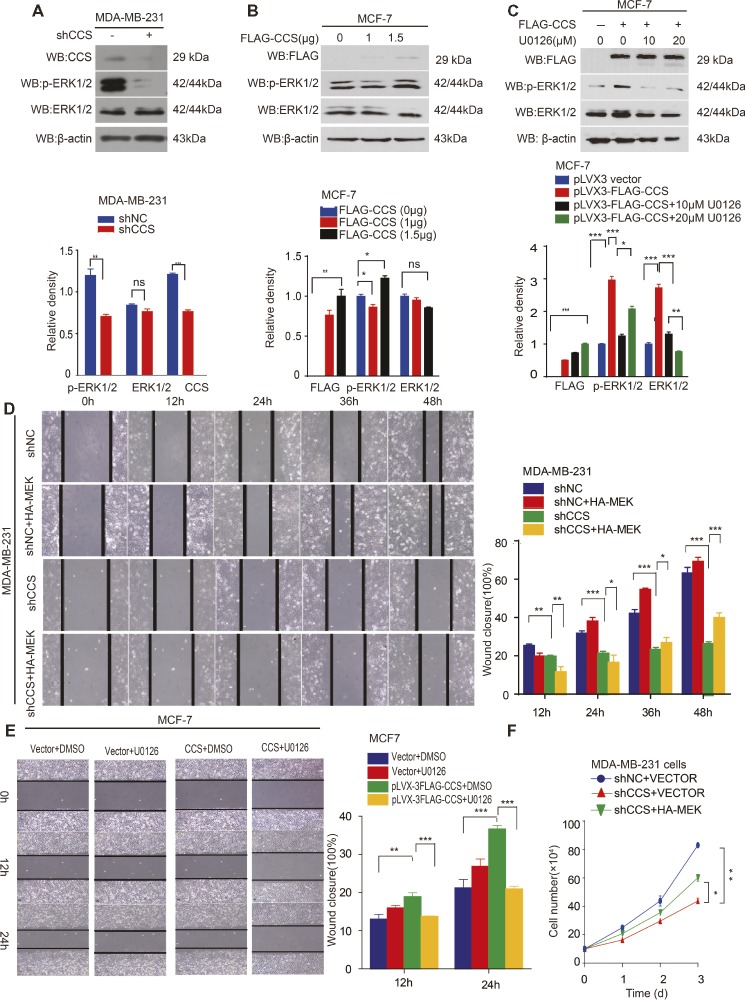
CCS promotes breast cancer cell migration and cell proliferation *via* ERK1/2 activity. **(A)** Phosphorylated ERK1/2 and total ERK1/2 levels were determined in CCS knockdown MDA-MB-231 cells by western blotting. **(B)** Phosphorylated ERK1/2 and total ERK1/2 levels were determined in CCS overexpressing MCF-7 cells by western blotting. **(C)** Phosphorylated ERK1/2 and total ERK1/2 levels were determined in CCS overexpressing MCF-7 cells treated with increasing concentrations of U0126 for 12 h by western blotting. **(D)** Cell migration in CCS knockdown and control MDA-MB-231 cells as determined by wound healing assay when overexpressing exogenous HA-tagged MEK. **(E)** Cell migration in CCS overexpressing and control MCF-7 cells treated with U0126 was determined by wound healing assay. The modified migration assay was evaluated by calculating the ratio of the cell numbers through the chamber or wound closure after the wound healing assay. **(F)** Cell proliferation was determined by cell number counting assays in CCS stable knockdown MDA-MB-231 cells with overexpression of exogenous HA-tagged MEK. All results performed above are presented as mean ± SD from three independent experiments. *p < 0.05; **p < 0.01; ***p < 0.001, ns not significant.

Activation of survival signaling has been shown to play an essential role in tumor development (Baud and Karin, 2001). Several studies have demonstrated that the MAPK/ERK signaling pathway is activated in cancer cells to promote cancer cell proliferation, migration, and invasion (Rajalingam et al., 2005; El Touny et al., 2014). Therefore, we examined whether MAPK/ERK signaling is involved in CCS mediated cell proliferation and migration. To test this hypothesis, we examined the ERK1/2 and MEK1/2 activity in CCS knockdown MDA-MB-231 cells. Western blotting shows that the activity of ERK1/2 was drastically decreased in CCS knockdown MDA-MB-231 cells (Figure 4A). Additionally, overexpression of FLAG tagged CCS increased the activity of ERK1/2 in MCF-7 cells (Figure 4B), but the increased activity of ERK1/2 was blocked in MCF-7 with ERK inhibitor U0126 (Figure 4C). To validate the role of MAPK signaling in the process of CCS-induced migration and proliferation in breast cancer cells, we first reactivated ERK by transfecting exogenous HA tagged MEK into MDA-MB-CCS-KD cells. As expect, the replenishment of MEK in MDA-MB-231-CCS-KD cells could partially rescue the capability of migration in MDA-MB-231-CCS-KD cells due to the reactivation of ERK1/2 (Figure 4D). Secondly, we demonstrated that inhibition of MEK with U0126 treatment inhibited CCS-induced cell migration (Figure 4E). Thirdly, overexpression of MEK in MDA-MB-231-CCS-KD cells partially rescues the decreased cell proliferation in CCS knockdown MDA-MB-231 cells (Figure 4F). These results suggest that activation of the MAPK/ERK pathway is essential for the CCS-promoted migration abilities and cell proliferation of breast cancer cells.

### CCS Activates MAPK/ERK Signaling *via* ROS

**Figure 5:**
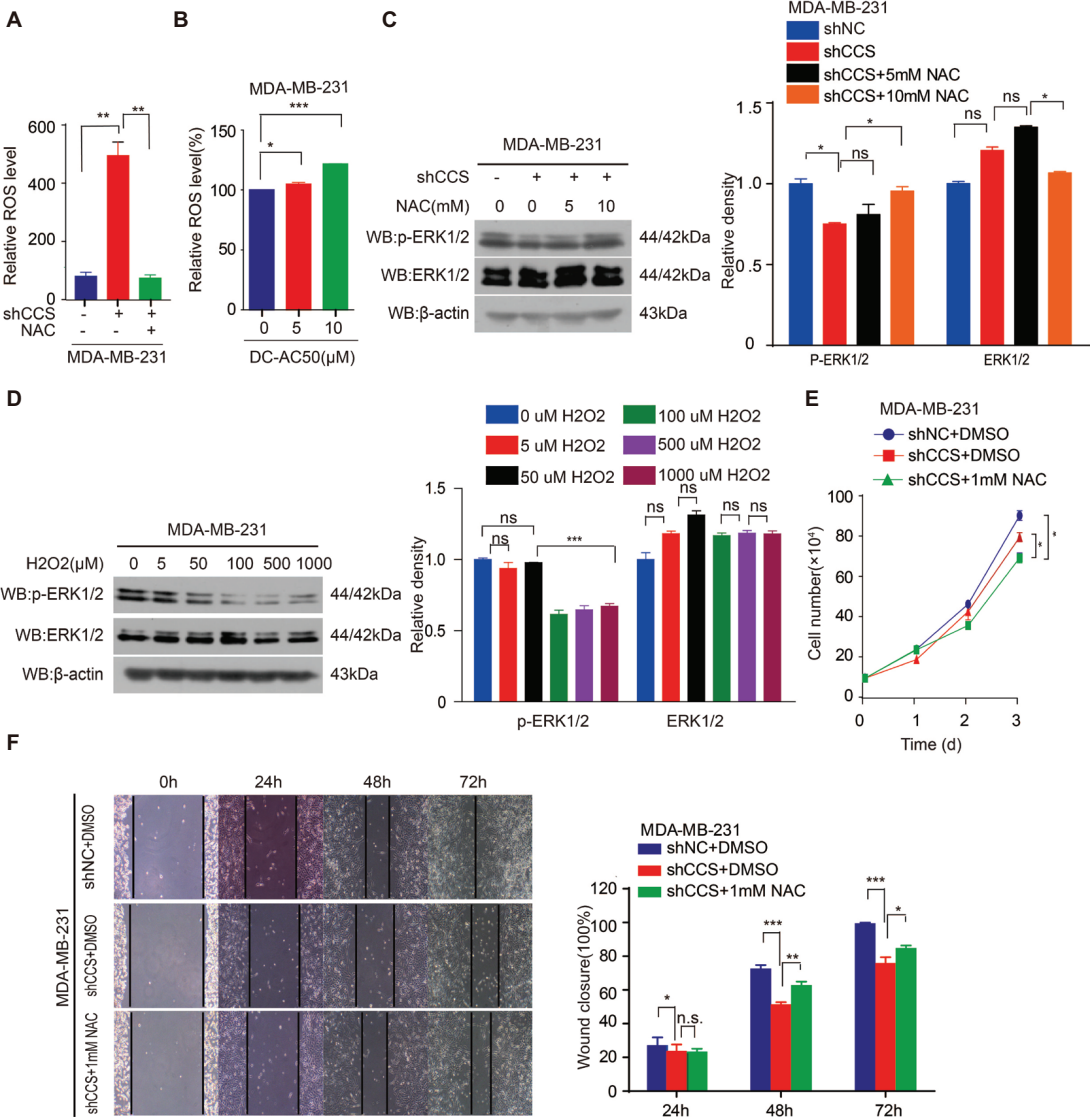
CCS promotes breast cancer cell migration and cell proliferation via ERK1/2 activity mediated by ROS. **(A)** Knockdown of CCS increased ROS level in MDA-MB-231 cells, which was rescued by treatment with 1 mM NAC. **(B)** Treatment with DC_AC50 (5, 10 μM) induced ROS elevation in MDA-MB-231 cells. **(C)** Western blot analysis of total and phosphorylated ERK1/2 levels. β-actin was used as a loading control. Reduced ERK1/2 activity by CCS knockdown was rescued by treatment with NAC (5, 10 mM) for 12 h. **(D)** H_2_O_2_ significantly abolished ERK1/2 activity in MDA-MB-231cells after 12 h. **(E)** Cell proliferation assays showed that NAC (1 mM) treatment partially rescued the decreased cell proliferation in CCS knockdown MDA-MB-231 cells. **(F)** Wound healing assays showed that NAC (1 mM) treatment partially rescued the decreased cell migration in CCS knockdown MDA-MB-231 cells. All results performed above are presented as mean ± SD from three independent experiments. *p < 0.05; **p < 0.01; ***p < 0.001, ns: not significant.

The inhibition of CCS leads to increased ROS levels. Thus, we hypothesized that CCS regulates the activity of ERK1/2 through ROS. To test this hypothesis, we examined ROS levels in MDA-MB-231 cells treated with CCS shRNA or DC_AC50. Indeed, we found that knockdown or inhibition of CCS significantly increases the cellular ROS levels (Figures 5A,B), while the increased ROS was blocked by treating cells with antioxidant N-Acetyl-L-cysteine (NAC; Figure 5A). In addition, we also observed that NAC abrogates the decreased activity of ERK1/2 in CCS knockdown MDA-MB-231 cells (Figure 5C). Consistently, we also found that H_2_O_2_ impaired phosphorylation of ERK1/2 in a dose-dependent manner but did not affect the total expression level of ERK1/2 (Figure 5D). Finally, we found that NAC could rescue the decreased cell proliferation and migration of MDA-MB-231 CCS knockdown cells (Figures 5E,F). These results further support the idea that inhibition of CCS induces a ROS overload, which impairs MAPK/ERK signaling to attenuate cancer cell proliferation. The combined results presented here also establish CCS as a viable anticancer target and copper trafficking as a new pathway for future therapeutic development.

## Discussion

**Figure 6:**
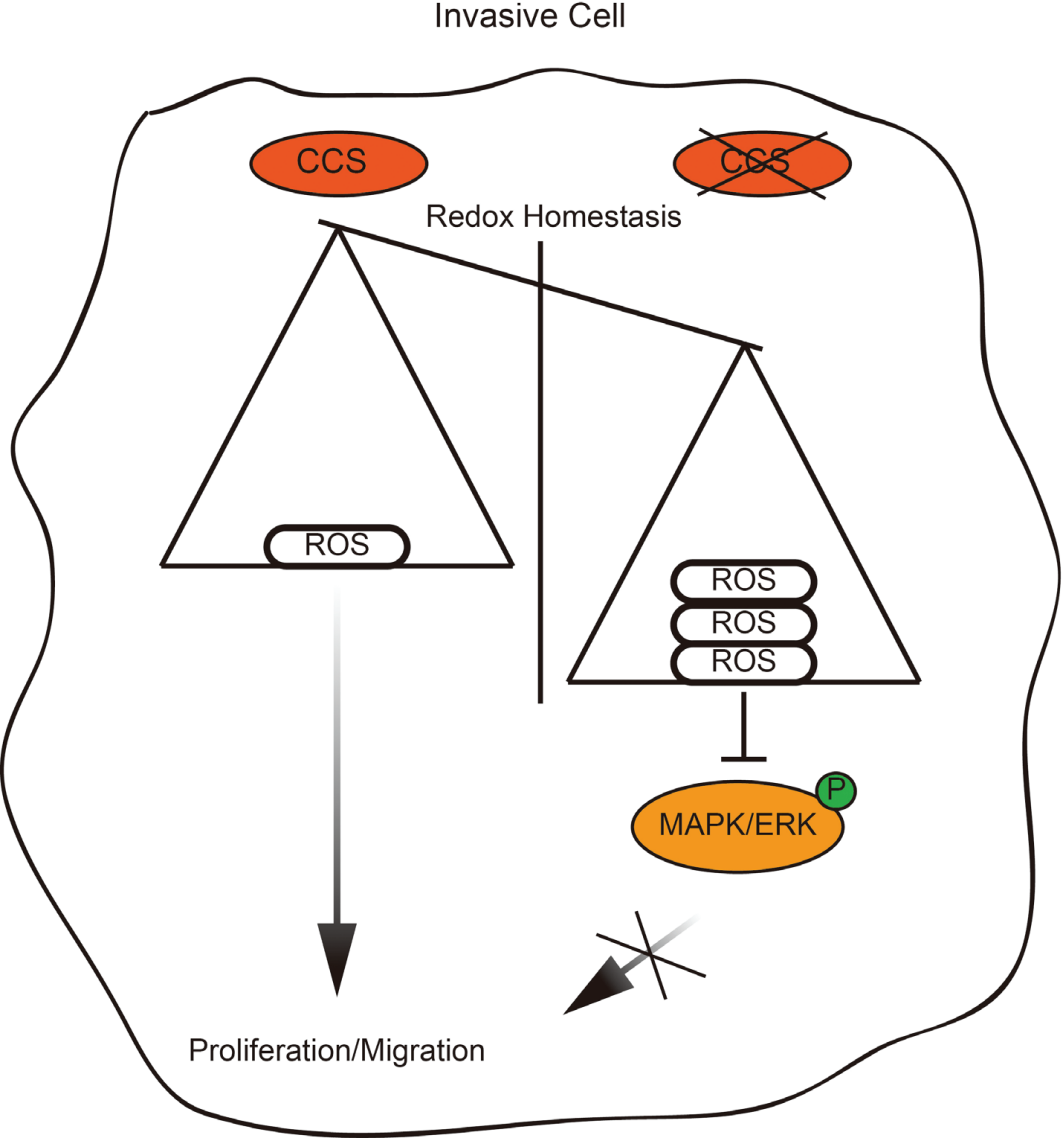
Proposed working model. Schematic model shows that the role and mechanism of CCS in promoting breast cancer cell proliferation and migration.

Rapid cellular growth and migratory abilities play a crucial role in tumorigenesis and metastasis, which have been recognized to be associated with ROS levels (Aykin-Burns et al., 2009; Doskey et al., 2016). Those cells that survive oxidative stress stand a good chance to have acquired adaptive mechanisms to counteract the potential toxic effects of elevated ROS and to promote cell-survival pathways (Irmak et al., 2003). CCS, a co-enzyme of SOD1, is a critical component of the oxidation–reduction system in cancer, and its differential expression in different types of breast cancer suggests a relationship between CCS and cancer cell growth and migration (Figure 6). However, the link between CCS-activated ROS and the occurrence and development of tumors is still in its infancy.

In this study, we utilized MDA-MB-231 cells (triple-negative breast cancer) and MCF-7 cells (estrogen receptor positive breast cancer) as human cell line models and identified a novel function and mechanism of CCS in facilitating breast cancer cell proliferation and migration. This novel mechanism provides a link between oxidative metabolism and survival signaling. Wang et al. reported that inhibition of CCS leads to a selective suppression of cancer cell proliferation (Wang et al., 2015). Consistent with this, we found that knockdown of CCS significantly reduced cell proliferation in MDA-MB-231 cells but not MCF-7. Interestingly, we revealed a novel function of CCS in regulating migration of breast cancer cells by transwell and wound healing assays. In all, we show that CCS not only plays a vital role in cell proliferation, but it also drives breast cancer migration.

Previous evidence has shown that CCS serves as a co-enzyme of SOD1 to activate its catalytic activity, which is a critical component of oxidation–reduction system (Suzuki et al., 2013b). Since ROS associated oxidative stress has been proven to play important roles in several cancer types and served as promising target for therapy (Perše, 2013; Sosa et al., 2013), we hypothesized that dysregulated ROS levels provide a second signal for CCS-induced proliferation and migration in breast cancer cells. In our study, we showed that knockdown or inhibition of CCS led to increased total ROS levels in MDA-MB-231. ROS overload blocks the activation of the MAPK/ERK pathway, which plays a critical role in tumor formation and progression (Berger et al., 2017; Mayo et al., 2017). By mimicking oxidative stress with H_2_O_2_ treatment, we were able to suppress the phosphorylation level of ERK1/2, which could be reversed upon treatment with antioxidant NAC. Furthermore, we found that the activation of MAPK/ERK pathways was essential for CCS-induced cell proliferation and migration. Treatment of MCF-7 with U0126-EtOH, a highly selective ERK kinase inhibitor, diminished CCS-induced migration. Conversely, overexpression of MEK enhanced the phosphorylation level of ERK1/2 and partially rescued migration in CCS knockdown MDA-MB-231 cells.

In summary, CCS-mediated ROS decreases the activation of ERK1/2, resulting in attenuation of cell proliferation and migration. Thus, CCS may be a therapeutic strategy to suppress tumor growth and metastasis.

## Author Contributions

RL and YL performed and analyze all the experiments. XZ and JW drafted the work an provided intellectual content. LL and CS edited the language and figures. CS, SL and SZ designed the study and wrote the manuscript.

### Conflict of Interest Statement

The authors declare that the research was conducted in the absence of any commercial or financial relationships that could be construed as a potential conflict of interest.

## Abbreviations

AMP: Adenosine Monophosphate
ATP: Adenosine Triphosphate
CCS: Copper Chaperone for Superoxide Dismutase
ERK: Extracellular Regulated Protein Kinases
MAPK: Mitogen-Activated Protein Kinase
NAC: N-Acetyl-L-cysteine
ROS: Reactive Oxygen Species
PCR: Polymerase Chain Reaction
PEI: Polymine
PVDF: Polyvinylidene Fluoride
qRT-PCR: Real-time Quantitative Reverse Transcription-PCR
SDS-PAGE: Sodium Dodecyl Sulfate Polyacrylamide Gel Electropheresis
ShRNA: Short Hairpin RNA
SiRNA: Small Interfering RNA
SOD1: Superoxide Dismutase
TCGA: The Cancer Genome Atlas.
